# Selective Attention to Task-Irrelevant Emotional Distractors Is Unaffected by the Perceptual Load Associated with a Foreground Task

**DOI:** 10.1371/journal.pone.0037186

**Published:** 2012-05-23

**Authors:** Catherine Hindi Attar, Matthias M. Müller

**Affiliations:** 1 Institute of Psychology, University of Leipzig, Leipzig, Germany; 2 Department of Systems Neuroscience, University Medical Center Hamburg-Eppendorf, Hamburg, Germany; University of Manchester, United Kingdom

## Abstract

A number of studies have shown that emotionally arousing stimuli are preferentially processed in the human brain. Whether or not this preference persists under increased perceptual load associated with a task at hand remains an open question. Here we manipulated two possible determinants of the attentional selection process, perceptual load associated with a foreground task and the emotional valence of concurrently presented task-irrelevant distractors. As a direct measure of sustained attentional resource allocation in early visual cortex we used steady-state visual evoked potentials (SSVEPs) elicited by distinct flicker frequencies of task and distractor stimuli. Subjects either performed a detection (low load) or discrimination (high load) task at a centrally presented symbol stream that flickered at 8.6 Hz while task-irrelevant neutral or unpleasant pictures from the International Affective Picture System (IAPS) flickered at a frequency of 12 Hz in the background of the stream. As reflected in target detection rates and SSVEP amplitudes to both task and distractor stimuli, unpleasant relative to neutral background pictures more strongly withdrew processing resources from the foreground task. Importantly, this finding was unaffected by the factor ‘load’ which turned out to be a weak modulator of attentional processing in human visual cortex.

## Introduction

To date, there is abundant evidence that emotional stimuli receive prioritized processing due to their inherent significance for adaptive behavior and survival (cf. [1], [2], [3], [4]). For example, highly arousing emotional scenes [5], [6] or phobic stimuli [7] have been shown to facilitate sensory processing in visual cortex. Contemporary models of selective attention such as the biased competition model of attention [8] posit that multiple stimuli compete for limited sensory processing resources. An emotional stimulus which occurs with a concurrently presented neutral stimulus for processing may dissolve this competition by biasing attention toward its emotionally significant information at the expense of the other stimulus. Indeed, a large number of different experimental paradigms, including visual search and dot probe tasks have shown, that subjects exhibit reduced search time and faster responses to threat-related relative to neutral stimuli [3], [9]. This affective bias was further confirmed by findings from event-related potential (ERP) studies in which enhanced early posterior negativity (EPN) between 200 ms and 300 ms after stimulus onset and increased late positive potentials (LPPs) most pronounced between 400 ms and 700 ms post-stimulus onset for emotional compared to neutral stimuli were observed [10], [11], [12].

In the field of visual attention, one important factor that is proposed to determine the selection of perceptual information is the perceptual load imposed by a foreground task. According to Lavie's (1995) original concept of perceptual load, attentional selection of a given stimulus depends on the attentional demands of the foreground task. If the task requires little attentional demands to make a perceptual discrimination (i.e. low load) enough resources are left to process also task-irrelevant features. However, if attentional demands are increased under conditions of high perceptual load, fewer attentional resources are left to process other task-irrelevant stimuli. Substantial amounts of research have ascertained that irrelevant distractors can produce significant interference providing that the attentional load of the relevant task is low [13], [14]. Adapting this framework for emotion research, evidence from behavioral [15], [16] and functional magnetic resonance imaging (fMRI) data [17] has suggested that the processing of emotional stimuli requires some degree of attention, too. In contrast, findings from recent fMRI studies [18], [19] did not confirm such interactive processes between attentional load and emotional stimulus salience. Instead, both factors are assumed to have independent influences on visual processing probably mediated by different neural sources [20].

Previous electrophysiological studies investigating the effect of perceptual load manipulations on the processing of task-irrelevant emotional distractors observed larger amplitudes of the N1/P2 components at parietal-occipital electrode sites under low load, but not under high load [21]. Schupp et al. [22] reported a similar emotion by load interaction, which was reflected in the EPN component. Additional source estimations revealed that the differential processing of pleasant and unpleasant picture content was primarily modeled by sources over occipito-temporo-parietal regions [22]. The finding that increased attentional load can abolish cortical responses associated with emotional processing was recently challenged by an ERP study, which observed sustained emotion effects irrespective of attentional load as reflected in the LPP component [23]. The discrepant results derived from ERP studies might be attributed to noticeable differences between the experimental designs. The manipulation of load used to divert attention from the emotional distractors ranged from delayed line discrimination tasks [21] over line counting tasks [22] to arithmetic subtraction tasks [23]. A recent magnetoencephalographic (MEG) study whose task design adopted the original definition of perceptual load [13] observed emotional distractor processing that varied as a function of time [24]. Specifically, this study showed early gamma band activity in the amygdala in response to emotional stimuli (40–140 ms) that was unaffected by load while late responses between 280 ms and 410 ms were modulated by load [24].

Recently we showed that competition for processing resources in visual cortex between a task and emotional images follows a distinct time course with different processing stages [25], [26]. In these studies, subjects saw flickering dots that elicited the steady-state visual evoked potential (SSVEP) which were superimposed on IAPS pictures. The SSVEP is the oscillatory potential field generated by visual cortical neurons in response to a flickering stimulus that indexes neural activity related to stimulus processing. The amplitude of the SSVEP is substantially increased when the driving stimulus is attended [27], [28], [29], thereby providing a sensitive and direct neuronal measure of the time course of attentional resource allocation [26], [30], [31]. Subjects were instructed to attend to these flickering dots and to detect rare coherent motion events. SSVEP amplitudes elicited by the dots were significantly more reduced when emotional compared to neutral IAPS pictures were concurrently presented in the background of the screen. Importantly, that reduction was not across the entire length of a trial, but was restricted to a time window between 400 ms to 1000 ms after the onset of the pictures. Behavioral data paralleled electrophysiological findings by a decrease in hit rates for emotional distractors in almost the same time window [25]. However, one possible limitation of our studies was that we did not manipulate perceptual load. Thus, despite overall hit rates of 60 to 70% indicating high task demands, we were not able to differentiate between the influence of emotional distractors under conditions of low and high load.

### Objective

To directly test the effect of perceptual load on emotional distractors, we presented a symbol stream at fixation which required subjects to either perform a relatively easy symbol detection task (low load) or a demanding symbol discrimination task (high load). In addition, task-irrelevant neutral or unpleasant pictures from the International Affective Picture System (IAPS) [32] were concurrently presented in the background. Different from our previous studies, both task- and distractor stimuli flickered at different frequencies to elicit distinguishable SSVEPs. Notably, using flickering background images goes at the expense of temporal resolution given that with each onset of the image, emotional content might be extracted. In fact, several studies reported similar short stimulus presentation times that were sufficient for emotional content extraction [33], [34], [35], [36]. However, it is important to note that Ferrari and colleagues [37] have recently shown that massed picture repetition did not crucially affect differences between emotional and neutral pictures as reflected in the LPP.

Based on these findings we hypothesized that if perceptual load has an influence on emotional distractor processing we would expect that SSVEP amplitudes elicited by unpleasant images are enhanced only under conditions of low task demands (i.e. low perceptual load). Consequently, we would further predict that emotional distractors compared to neutral ones more strongly reduce SSVEP amplitudes directed to the foreground task.

## Materials and Methods

### Ethics Statement

All subjects provided written informed consent prior to the study which was approved by the ethics committee of the University of Leipzig.

### Participants

Fifteen healthy, right-handed subjects (11 female) with a mean age of 23.8 years (standard deviation [SD] = 2.9 years) and normal or corrected to normal visual acuity participated in the experiment. All subjects received either a small financial bonus (6 Euros per hour) or credit points for participation. The instruction manual contained two examples of the forthcoming picture material consisting of a household scene for the neutral condition and a severe injury scene for the unpleasant condition. None of the subjects refused the participation thereupon.

### Stimuli

**Figure 1:**
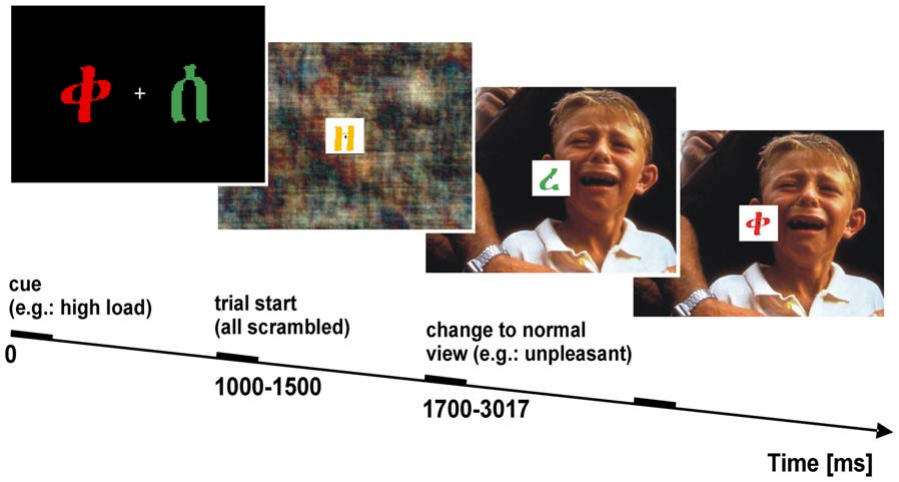
Schematic illustration of a trial sequence. Each trial (here: high perceptual load condition) started with a cue (1000–1500 ms) that indicated the two symbols, which had to be detected within the symbol stream. Low perceptual load required subjects to respond to the blue color of these symbols, high perceptual load involved a discrimination task (respond when one symbol is printed in red or the other symbol is printed in green). Symbols were presented at a stimulation frequency of 8.5 Hz while the underlying IAPS picture was flickering simultaneously at 12 Hz. Stimulation started with the scrambled version of the IAPS picture (see Methods for scrambling procedure) that changed between 700–1517 ms to normal view of either a neutral or an unpleasant image (here: unpleasant). Inter-trial intervals lasted 700 ms and a fixation cross was present throughout the experiment.

Visual stimuli were presented using Cogent (www.vislab.ucl.ac.uk/Cogent2000), a MATLAB toolbox allowing precise timing and synchronization with the EEG system, and shown on a 19-inch computer screen (CRT) at a viewing distance of 80 cm and a refresh cycle of 60 Hz. Ten different unfamiliar symbols of five different colors (blue, red, green, yellow and purple) embedded in a white square (1.8°×1.8°) formed the symbol stream and were rapidly presented at a frequency of 8.6 Hz at the center of the screen. The symbol stream was overlaid on a task-irrelevant neutral or unpleasant IAPS picture (12.8°×9.4°) that flickered at a rate of 12 Hz (see Figure 1). A small gray fixation cross was continuously present at the center of the screen throughout the experimental trials. For the neutral and unpleasant picture sets, 40 pictures were selected based on the normative valence and arousal ratings provided by the IAPS set. According to these ratings, mean valence and arousal values for the neutral picture set were 4.97 and 4.49, and 2.71 and 5.80 for the unpleasant set. To control for perceptual complexity, we carefully scanned the picture contents to provide for a comparable relation of simple figure/ground versus complex scene stimuli between the emotional and the neutral picture set. For instance, in case of the neutral picture set, several complex scenes (e.g. office scenes) were included instead of portrait pictures or pictures with simple household objects with typically less complex figure-ground segregation. To further hedge our subjective measures of image complexity we also used JPEG size as an automated measure of perceptual complexity which has been shown to highly correlate with human estimates of visual complexity [38]. Importantly, JPEG size (mean ± SE; neutral: 462±44, unpleasant: 364±34) did not significantly differ between the two picture sets (t_39_ = 1.91, p>0.05). Last, we made sure that the gist of the picture scene was not hidden behind the foveally presented symbol stream.

Similar to our previous experiment [25] we presented scrambled versions of the pictures at trial onset that served as a baseline measure. Scrambling of pictures was performed by a Fourier transform, yielding the amplitude and phase components of each image. Before rebuilding the image with an inverse Fourier transform, the original phase spectrum was replaced with random values, keeping the amplitude spectrum of the image unaltered. The resulting pictures were characterized by equal global low-level properties of the original image (luminance, spectral energy), while any content-related information was deleted. Luminance of the experimental display was 55.5 cd/m^2^ on average for the symbol stream and 22.6 cd/m^2^ on average for the IAPS images. Mean luminance of pictures did not differ between picture sets.

### Experimental Procedure

As depicted in Figure 1, each trial started with a display of a cue (1000–1500 ms duration) that indicated whether subjects had to perform the high load or low load task. Sequence of task conditions was randomized and thus unpredictable for our subjects. Subjects were assigned two target symbols and were instructed to detect the occurrences of those targets within a trial. During each trial, a total of 10 different symbols in a stream of 35 symbols were presented. For the low load task, subjects were instructed to press a response button if they saw one of two symbols that were printed in the color blue (pop-out detection). For the high load task, subjects had to discriminate between the two identical symbols as in the low load task and had to press the response button whenever the one symbol was printed in red or the other symbol was printed in green. The responding hand was changed halfway through the experiment. Between zero and four targets were embedded in the symbol stream of a single trial. After cue presentation, both the symbol stream and the scrambled version of an IAPS picture started flickering at its respective frequency. Thus, in each cycle of the corresponding frequency each symbol was 67 ms ‘on’ and 50 ms ‘off’ while the background picture was 33 ms ‘on’ and 50 ms ‘off’.

To prevent temporal expectation effects, the scrambled background picture changed to either a neutral or unpleasant picture within a variable time interval between 700 to 1517 ms. Accordingly, as each trial lasted for 4083 ms in total, concrete picture presentation time was between 2566 ms and 3383 ms (see Figure 1). Each trial was followed by an inter-trial interval of 700 ms. The entire experiment consisted of 8 successive blocks of 40 trials each. At the end of each block feedback upon task performance was provided. All IAPS pictures were randomly presented 2 times throughout the experiment (no repetition within the next three pictures), resulting in 80 trials per experimental condition. Pseudorandom trains of symbols were created for each trial, with the same number of targets in the low and high load conditions that were equally distributed across the entire trial length to maintain attention to the task. Similar to recent studies [39], stimulus sequences of the symbol stream were constructed such that they could be used in either load condition, effectively equating stimulus characteristics between conditions so that load only varied through the assignments of different targets. Subjects were instructed to always attend to the task at fixation while the concurrently presented background picture was task-irrelevant and to be ignored.

Before the experiment, subjects performed several practice trials until they became familiar with the task and reached minimum target detection rate of 60% in the high load condition. These trials involved IAPS pictures which were not used in the experimental trials. After EEG recording, participants completed subjective ratings regarding valence and arousal for each IAPS picture presented during the EEG session with a computer version of the Self-Assessment Manikin rating scales (SAM [40]).

### EEG recording and analysis

EEG was recorded from 64 Ag/AgCl electrodes mounted in an elastic cap at a sampling rate of 256 Hz using a BioSemi ActiveTwo amplifier system (BioSemi, Amsterdam, The Netherlands). Electrode layout followed the extended international 10–20 system. Additional electrodes located at the outer canthi and above and below the left eye were used to determine the horizontal and vertical electrooculogram (EOG). The picture stream and the symbol stream flickered at different frequencies which allowed us to extract distinguishable SSVEPs. Both stimulus streams were phase synchronized at the moment when the scrambled version of the image changed to a concrete image. From that time point we extracted epochs starting 1000 ms before and lasting until 2800 ms after picture change. Next, the mean and any linear trend was subtracted from each epoch. Epochs containing blinks and eye movements were rejected from further analysis. Artifacts such as noisy electrodes were corrected using a combination of channel approximation and epoch exclusion based on statistical parameters of the data with the ‘statistical control of artifacts in dense array EEG/MEG studies’ (SCADS) [41]. 96% of trials were retained and their number did not differ significantly between conditions. Data were then algebraically transformed to average reference. Subsequently, we averaged all artifact free trials for each subject and channel separately for each experimental condition.

To determine the appropriate electrodes for further analysis, iso-contour voltage maps of the 8.6 Hz (symbols) and 12 Hz (pictures) SSVEP amplitudes were calculated by means of Fourier transform of a time window from 200 ms before to 2000 ms after picture change and subsequently averaged across either the factor valence or the factor load. The appropriate electrodes were then selected based on the difference between iso-contour voltage maps related to the factors valence (i.e. emotional minus neutral) and load (i.e. high load minus low load). For each electrode, SSVEP amplitude peaks of the four experimental conditions were analyzed separately at the two stimulation frequencies. Specifically, these voltage values entered repeated-measures ANOVAs comprising the factors of *Electrode* (selected on the basis of SSVEP difference voltage maps), *Valence* (neutral, unpleasant pictures) and *Load* (low, high), for the 8.6 Hz (symbol) and 12 Hz (picture) streams, respectively. Greenhouse-Geisser adjustments were performed whenever appropriate. Single comparisons between experimental conditions were calculated by means of paired t-tests.

### Behavioral data analysis

Button presses within a time window of 200 ms to 800 ms post target-onset were counted as a correct response. Behavioral data was analyzed by calculating the mean target detection rates and reaction times for each subject within 3 time bins (baseline, 1st second after picture change, 2nd second after picture change). Repeated-measures ANOVAs were calculated for the target detection rates and reaction times by crossing the within-subject factors *Valence* (neutral, unpleasant), *Load* (high, low) and *Time* (3 time bins). Differences between conditions were tested for the three time bins by paired t-tests. Differences between the mean valence and arousal ratings for the neutral and unpleasant pictures obtained by the SAM scale were also tested by paired t-tests.

## Results

### Behavioral results

SAM ratings showed significant differences between valence and arousal ratings for the two picture categories. Unpleasant pictures were rated as less pleasant (mean pleasure rating 2.09, SD = 0.60) than neutral pictures (mean pleasure rating 5.52, SD = 0.63; t_14_ = 17.02, p<0.0001). In the same vein, unpleasant pictures were rated as significantly more arousing (mean arousal rating 6.45, SD = 1.00) than neutral pictures (mean arousal rating 2.77, SD = 0.95; t_14_ = −13.88, p<0.0001).

**Figure 2:**
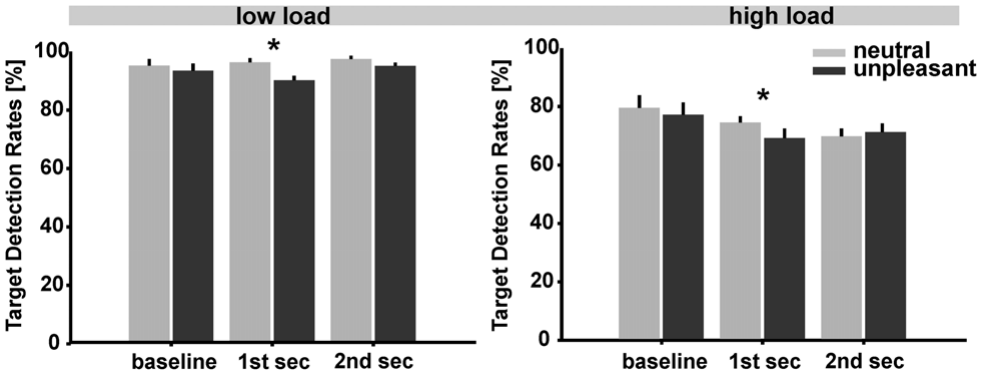
Target detection rates. Mean target detection rates in percent and corresponding standard errors for 3 successive time windows of one second each (at baseline before picture change, 1^st^ second and 2^nd^ second after picture change) averaged across all subjects for neutral (light gray bars) and unpleasant pictures (dark gray bars), separately for low load and high load conditions.

The repeated-measures ANOVA on target detection rates yielded a significant main effect of *Load* (F_1;14_ = 83.678, p<0.0001). Target detection rate was overall higher under the low load relative to the high load condition, thus indicating that task load was effectively manipulated (Figure 2). Importantly, a significant main effect was also observed for the factor *Valence* (F_1;14_ = 6.424, p<0.05). In a next step, we compared task performance in the context of unpleasant and neutral distractors within each time window. Under low load, target detection rates were generally lower for unpleasant compared to neutral distractors. This difference was statistically significant within the first time window (i.e. 1st second: t_14_ = 5.375, p<0.0001) and at trend level within the second window (i.e. 2nd second) following picture change (t_14_ = 1.920, p = 0.08). Likewise, under high load, target detection rates within the first second after picture change were significantly reduced for unpleasant relative to neutral distractors (t_14_ = 2.504, p<0.05, see also Figure 2).

A similar repeated-measures ANOVA on reaction times yielded a main effect of *Load* (F_1;14_ = 151.094, p<0.0001) with mean reaction times of 370±21.60 ms for the low and 435±27.32 ms for the high load condition. Thus, reaction times further confirmed the effectiveness of the load manipulation associated with the foreground task. Since no other main and interaction effects reached significance, no more single comparisons between conditions were conducted.

### SSVEP results

**Figure 3:**
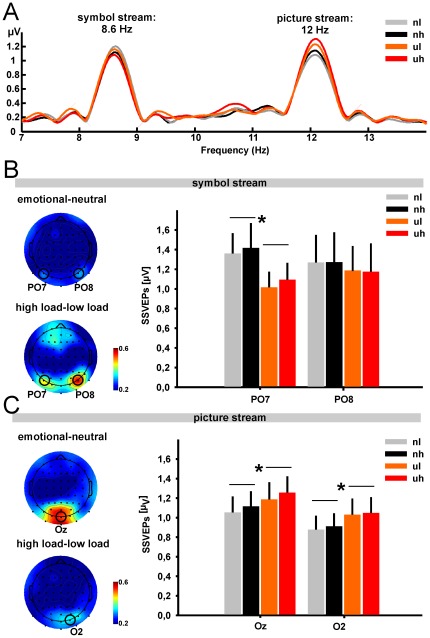
SSVEP amplitudes and iso-contour voltage maps. (A) Grand-average spectrum obtained by Fourier analysis of SSVEP waveforms for all four experimental conditions, i.e. neutral pictures and low load (light grey line), neutral pictures and high load (black line), unpleasant pictures and low load (orange line) and unpleasant pictures and high load (red line) averaged across occipital electrodes (PO8, OZ, PO7). Peaks are located at the two stimulation frequencies at which the symbol stream and the picture stream were presented. (B) Iso-contour voltage maps based on the difference between the topographical distribution of emotional minus neutral SSVEP amplitudes (upper left panel) and high load minus low load SSVEP amplitudes (lower left panel) at the stimulation frequency of the symbol stream (8.6 Hz). The right panel shows the grand-average SSVEP amplitudes and corresponding standard errors for the symbol stream and each experimental condition for the two electrodes indicated in the iso-contour voltage maps (PO7, PO8). (C) Iso-contour voltage maps based on the difference between the topographical distribution of emotional minus neutral SSVEP amplitudes (upper left panel) and high load minus low load SSVEP amplitudes (lower left panel) at the stimulation frequency of the picture stream (8.6 Hz). The right panel shows the grand-average SSVEP amplitudes and corresponding standard errors for the picture stream and each experimental condition for the two electrodes indicated in the iso-contour voltage maps (OZ, O2). Abbreviations: nl = neutral low load; nh = neutral high load; ul = unpleasant low load; uh = unpleasant high load.

Figure 3A depicts the grand-average SSVEP amplitude spectra from all four experimental conditions averaged across occipital electrodes (PO7, OZ, PO8). As can be seen in this figure, both stimuli elicited SSVEP amplitudes at about the same magnitude.

For the symbol stream, the repeated-measures ANOVA yielded a significant main effect of *Valence* (F_1;14_ = 5.560, p<0.05) and a significant *Electrode x Valence* interaction (F_1;14_ = 6.277, p<0.05). Follow-up paired t-tests on SSVEP amplitudes for unpleasant relative to neutral background pictures averaged across the factor load showed a significant reduction of SSVEP amplitudes for unpleasant relative to neutral distractors at electrode PO7 (t_14_ = 2.208, p<0.05) but not at the corresponding electrode PO8 on right-lateralized occipital sites (t_14_ = 0.169, p>0.5). Iso-contour voltage maps of differences between SSVEP amplitudes for the factors valence and load at the stimulation frequency of the symbol stream and the extracted SSVEP amplitudes at the selected electrodes are shown in Figure 3B.

Figure 3C depicts the difference iso-contour voltage maps of SSVEP amplitudes at the stimulation frequency of the picture stream. As these 12 Hz SSVEP amplitudes exhibit a more centrally located peak at electrode OZ compared to the topographical distributions that we observed for the 8.6 Hz SSVEP amplitudes of the symbol stream, the SSVEP signal was extracted from a different set of electrodes (i.e. OZ, O2). The repeated-measures ANOVA for the picture stream yielded a significant effect of *Electrode* (F_1;14_ = 9.665, p<0.001) and *Valence* (F_1;14_ = 6.019, p<0.05). As for the symbol stream, no main effects or interactions with the factor load were observed. Follow-up paired t-tests showed that SSVEP amplitudes averaged across the factor load were significantly enhanced in the presence of unpleasant distractors at both selected electrodes (OZ: t_14_ = −2.667, p<0.05; O2: t_14_ = 2.165, p<0.05).

### Control experiment: Effect of load on task-related SSVEP amplitudes without background images

Somewhat surprisingly to us, we did not find an effect of load on SSVEP amplitudes that were related to the rapid serial visual presentation (RSVP) of symbols. To test whether this was due to the combination of the symbol stream with IAPS pictures and/or was influenced by having two different stimulation frequencies that were partly spatially overlapping (i.e. at fixation), we presented the symbol stream yet without distracting IAPS pictures to a new group of 12 subjects (9 female; mean age 23.6 years; standard deviation [SD] = 2.5 years). The control experiment required subjects to perform the identical detection or discrimination task and comprised 8 blocks of 40 trials each, resulting in a total of 160 trials for the low and high load task condition, respectively. SSVEP amplitudes were quantified by means of a Fourier transform across occipital electrodes that were also used for the analysis in the main experiment (across occipital electrodes (PO7, OZ, PO8) and tested by means of paired t-tests. For behavioral data, differences in mean target detection rates and mean reaction times for high and low load were analyzed by means of paired t-tests.

**Figure 4:**
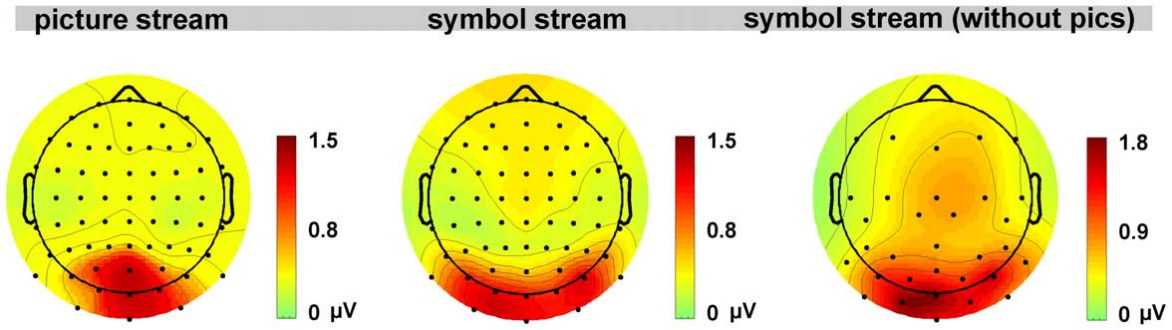
Iso-contour voltage maps. Iso-contour voltage maps of SSVEP amplitudes at the corresponding stimulation frequency averaged across experimental conditions for the main- and the control experiment where the identical symbol stream was presented yet without IAPS pictures in the background.

Identical to the main experiment, behavioral data showed significant higher target detection rates and slower reaction times for high load (mean target detection rates = 66.0%, SD = 12.2%; mean reaction times = 479 ms, SD = 30.5 ms) compared to low load (mean target detection rates = 96.3%, SD = 3.1%; t_11_ = 12.99; p<0.001; mean reaction times = 384 ms, SD = 32.4, t_11_ = −10.85; p<0.001), again confirming the effectiveness of task manipulation. In line with the main experiment, we observed no significant differences in SSVEP amplitudes when subjects performed either the high or low load task. In addition, voltage maps of the 8.6 Hz SSVEP amplitudes for the two symbol streams, averaged across conditions, did not differ between main- and control experiment but showed a more widespread, bilateral activation pattern when compared to the 12 Hz SSVEP distribution (Figure 4). Thus, the factor load has apparently no influence on SSVEP amplitudes and we can exclude that the failure to detect an effect of task load in the main experiment was linked to any attributes of the IAPS images such as stimulation frequency or physical features.

## Discussion

We presented our subjects an RSVP stream of unfamiliar symbols together with flickering unpleasant or neutral IAPS pictures. To manipulate load, subjects had to perform either an easy symbol detection or a demanding symbol discrimination task. Thus, similar to previous load studies (cf. [39]) the physical properties of the symbol stream were always identical. Although behavioral data confirmed successful load manipulation with reduced hit rates and prolonged reaction times in high load compared to low load trials, SSVEP amplitudes elicited by the symbol stream did not differ with load. In a control experiment, we were able to show that this lack was not due to the simultaneous presentation of IAPS pictures flickering at a different frequency in the background. Importantly, SSVEP amplitudes of the symbol stream were significantly attenuated in the presence of unpleasant compared to neutral background pictures. In contrast, SSVEP amplitudes elicited by the flickering IAPS pictures were significantly enhanced for unpleasant pictures. Identical to the symbol stream, no differences in amplitudes were found with respect to the factor load. Still, the presence of unpleasant distractors had a detrimental effect on task performance under both levels of load. This strongly supports our assumption that emotional stimuli are prioritized for processing, irrespective of load.

The present findings additionally support the findings of our previous studies [25], [26] where we used flickering dots that were superimposed upon emotional or neutral IAPS pictures and asked subjects to perform a coherent motion detection task. Contrary to these studies [25], [26], the present one was not designed to investigate the time course of competitive resource allocation to task stimuli and emotional distractors. Here, we capitalized on the advantage of the SSVEP technique to present each stimulus of interest at unique frequencies. Such frequency tagging allowed us to investigate competition for processing resources in early visual cortex in the frequency domain while stimuli competed for these resources. Thus, we have the unique possibility to directly measure neural responses in the visual cortex to both task- and distractor stimuli. However, as a detriment of the SSVEP, it is not able to detect transient attention or emotion effects at certain stages of stimulus processing as reflected in specific components of the ERP. Thus, we can not exclude that perceptual load might have affected very early components of the visual evoked potential. In fact, ERP studies have recently reported load effects already at the stage of the C1 component [42], which peaks around 60 ms to 90 ms and is commonly considered to reflect the first volley of sensory information reaching V1 [43]. However, the authors of this study [40] used non-emotional distracting material (multiple line patterns) and despite other reports of C1 modulations as a function of emotional stimulus content [44], [45], direct evidence for such an early attentional load effect in combination with emotional distractors is lacking. To date, there are few other ERP studies, which directly examined load by emotion interactions [11], [22], [23]. Findings from these studies [11], [22], [23] revealed both short-latency (P1, EPN) as well as sustained longer-latency (LPP) ERP emotion effects that were unaffected by task-related load manipulations. However, using a feature-based attentional manipulation, a recent ERP study observed emotion effects on the EPN component that were strongly attenuated under conditions of high load [22]. Notably, studies considerably differ with respect to stimuli, task manipulation and presentation mode, which makes a comparison of findings rather difficult. At least, the ERP results support the idea that automaticity, here in the context of emotion-related information processing, is not an all-or-none concept [46] but depend on several factors (e.g. specific forms of processing load, spatial arrangement of task- and distractor stimuli).

In view of ample evidence for effects of selective attention on the steady-state signal (cf. [27], [30], [47], [48]) our finding that perceptual load did not affect the SSVEP amplitudes in the present study was unexpected. These previous SSVEP studies usually involve paradigms in which SSVEP amplitudes were compared between attended and unattended flickering stimuli [28], [29], [30]. In the present study design however, selective attention was always directed to the foveally presented task-relevant stimuli. Thus, the effect of perceptual load which is supposed to determine the amount of attentional resources demanded by the task at hand might be too subtle to elicit modulations in the SSVEP signal. An alternative explanation might be that the effect of attention and top-down biasing signals triggered by the factor of perceptual load only affect higher order cortical visual areas [49] which are not modulated by the visual steady-state signals recorded in the present study.

As depicted in Figure 4, topographical distributions of the 8.6 Hz SSVEP amplitudes of the two symbol streams showed a more widespread, bilateral activation pattern compared to the topographical distribution of the 12 Hz SSVEP amplitudes of the picture stream which strongly affected central occipital sensors around electrode OZ. This difference in activation pattern might be related to differences in the size of the flickering stimuli with the background pictures being more than 35 times bigger than the symbols. It could also be the case that our task, which required subjects to detect and discriminate colored symbols has primarily affected more anterior, lateral-occipital regions including color region V4. This would also explain the bilateral amplitude peaks at electrodes PO7 and PO8 where we observed maximum effects of load and emotion for the symbol stream.

We also observed some activation for high versus low load at frontal sensors. So far, the precise cortical sources contributing to this frontal distribution of the 8.6 Hz steady-state signal in the current experiment are unknown. However, as competitive interactions between multiple visual stimuli are known to manifest within extrastriate cortex [8] we focused our analyses on lateral-occipital electrodes where we observed strongest effects on the 8.6 Hz signal amplitude. Admittedly, competition might also integrate across neural systems and carry through to higher level association areas in frontal areas as well [50]. Thus, it is likely that the somewhat stronger frontal distribution for high versus low load conditions reflects task-related higher level cortical processing although this proposal is purely speculative.

Central to our study was the neural competition for resources between the two frequency-tagged stimulus streams. If unpleasant pictures capture and hold resources, then they should act as competitors, interfering with the processing of a concurrent stimulus. Indeed, by examining both attentional allocation and cost effects associated with task-relevant and task-irrelevant stimuli, we were able to demonstrate that unpleasant distractors are processed at the cost of the competing task-relevant stimuli. This finding was reflected in reciprocal effects on the SSVEP amplitudes elicited by both competitors. Contrary, a recent SSVEP study [51] reported enhanced SSVEP amplitudes to flickering angry faces for high-anxious subjects. Notably, SSVEP amplitudes elicited by a simultaneously flickering competitive face remained unaffected, which speaks against a resource sharing account. According to the authors [47], this result suggests that threatening faces may allocate additional resources which are not at the cost of a competing social stimulus. However, that study differed from the present one in using a passive viewing paradigm. Earlier work with SSVEPs [6] where spatial attention was explicitly directed to one of two simultaneously flickering IAPS pictures observed similar additive effects of attended cues and their emotional content but also showed facilitated cortical processing of affective cues in the non-attended hemifield. Thus, there is an important difference whether attention is explicitly manipulated or whether spontaneous resource sharing effects are measured in a passive viewing paradigm.

### Caveats

The present study used fix tagging frequencies for task- and distractor stimuli (i.e. 8.6 Hz and 12 Hz). The observed emotion effect on SSVEP amplitudes related to each of the stimulation streams may therefore not be generalized to other frequency bands. However, it is important to note that changing the flicker frequency of the symbol stream automatically changes the presentation time of each symbol. Thus, higher stimulation frequencies would have also increased the difficulty of the foreground task making comparisons between different frequencies barely feasible. Moreover, counterbalancing stimulation frequencies would have also resulted in a too long recording time which might have compromised subject's compliance. Importantly, we could demonstrate in two previous studies from our lab that (i) presentation rate did not influence task performance in a color attention task [48], and that (ii) SSVEP amplitudes within the alpha range showed similar sensitivity to effects of competition than frequencies outside the alpha range [52]. This, together with the observation that source localization of SSVEP generators was practically identical for a number of frequencies and presentation modes [47], [48], [53] makes it very unlikely, that the current findings would have been different with other stimulation frequencies.

Second, due to the lack of any load effects on task- and distractor related SSVEP amplitudes we conclude that perceptual load seems to be a weak modulator of attentional processing in extrastriate visual areas. As previously stated, the present study was not designed to study attention-emotion interactions at precise time scale. Thus, short-lived effects of load on early visual areas may not have been detected. In addition, the use of a trial-by-trial variation of load instead of a blockwise alternation might have also prevented load effects to establish. In a recent ERP study [22], IAPS pictures were superimposed with line drawings that were silently to be count and task load was varied between experimental blocks. In this more predictable paradigm, high task load attenuated emotion effects at the level of the EPN component. However, this study was limited with regard to isolating the perceptual and cognitive aspects of task load. Moreover, task performance was not found to differentiate between different levels of load.

In summary, the present SSVEP results strongly support the view that unpleasant pictures are preferentially processed irrespective of the perceptual load associated with a foreground task. By using a paradigm which enabled the direct measuring of task- and distractor related processing, the present study supports the notion that perceptual load compared to emotional valence seems to be far less important in biasing competition for processing resources between an attentional foreground task and distracting emotional material.
